# Amblyopia Treatment Outcomes Re-Audit, Comparing Current Outcomes to Those from the 2011–12 Audit

**DOI:** 10.22599/bioj.306

**Published:** 2023-12-20

**Authors:** Michelle Blyth, Sarah Bryant

**Affiliations:** 1 Royal Victoria Infirmary, Newcastle Upon Tyne, UK

**Keywords:** audit, amblyopia, atropine, patching, VA outcomes, proportion VA change, episode

## Abstract

**Aim:**

An audit of the effectiveness of amblyopia treatment in the Newcastle Eye Centre (NEC) to determine how current visual acuity (VA) outcomes compare to those found in the 2011–12 audit.

**Methods:**

A retrospective database review. VA outcomes of patients who had undergone treatment for anisometropic, strabismic and mixed amblyopia; discharged between 31.08.2016 – 01.09.19, were compared with VA outcomes found in the previous audit. The previous audit reviewed patients commencing amblyopia treatment during 1.1.11–31.12.12.

An unpaired T-test was used to assess if results were statistically significantly different to those found previously. Proportion of visual change from commencement to completion of treatment was calculated. The duration of episode from first visit to discharge, adverse events and percentage of patients who achieved acceptable visual outcomes following only six to eight weeks of occlusion, were also analysed.

**Results:**

Between 31.8.16 and 01.09.19, 1,100 patients were discharged, of which 174 had completed amblyopia treatment and fit the inclusion criteria for the audit. Results show no statistically significant difference between current and previous VA outcomes for each type of amblyopia. The majority of patients (60%) achieve a VA outcome of ≤0.250 (logMAR) in the amblyopic eye. This is comparable to the previous audit where 59% of patients achieved a VA outcome of ≤0.250. Most patients still achieve a level of VA which is equal or almost equal to the fellow eye following amblyopia treatment. Treatment is still completed within a two-year period for the majority of patients (62%). There was only one adverse event and this related to atropine occlusion. Only 18 out of the 174 (10%) patients showed that occlusion could be discontinued following just six to eight weeks of treatment.

**Conclusions:**

The treatment of amblyopia in the NEC is as successful as found in the previous audit and the current amblyopia treatment protocol remains effective. Only 10% of patients achieved the appropriate VA for amblyopia treatment to be ceased on their first return visit. This indicates that the follow-up length for patients undergoing amblyopia treatment could be extended beyond six to eight weeks without causing a detriment to VA outcome.

## Introduction

Amblyopia is defined as “a unilateral or bilateral decrease of vision which persists after correction of the refractive error and removal of any pathological obstacles to vision” (Ansons & Davis, 2014: 285). Amblyopia treatment aims to improve VA of the amblyopic eye by means of conventional occlusion (patching), optical penalisation or pharmacological penalisation with cycloplegic drugs (atropine). Regular clinical audits looking at the treatment of amblyopia are essential to provide a measure of effectiveness of the protocol and quality of the service provided. This in turn highlights where good practice exists and allows implementation of changes where necessary.

The aim of this audit was to assess final VA outcomes of children who have recently undergone amblyopia treatment in the Newcastle Eye Centre (NEC), and compare to a previous audit to determine whether similar results are achieved following only one minor change to the amblyopia protocol. This change was the introduction of weaning from patching, whereby patients who have undergone more than three hours of occlusion daily taper down to two hours daily for two weeks before ceasing altogether, in an attempt to reduce risk of recurrence (PEDIG, 2004).

The previous audit analysed final VA outcomes for patients commencing occlusion treatment during 1.12.11–31.12.12.

In the NEC, the amblyopia treatment protocol gives parents/guardians for all amblyopic patients over the age of three years the choice between atropine and patching as a first line of treatment. In line with published research, if atropine occlusion is chosen as the method of treatment the instillation is twice weekly (PEDIG, 2004) and if patching is chosen as the method of treatment the amount prescribed is three to six hours daily (PEDIG 2003; PEDIG 2003; Stewart et al 2007). The protocol includes:

- An 18-week period for refractive adaptation for those requiring a glasses prescription for all types of amblyopia (Moseley et al, 2002)
- Patient information leaflets for glasses and amblyopia are issued at the start of treatment (including an additional information leaflet for atropine if this is the chosen method of occlusion)
- Aims, risks and effects of treatment are outlined to the parents/guardians at the start of treatment
- Vision progress charts are used as a visual aid to demonstrate change in vision over time
- Patients with amblyopia ≥0.500 are reviewed by the same orthoptist where possible, from previous experience that continuity can improve compliance and co-operation in some patients
- A six to eight week follow-up is arranged with the orthoptist after amblyopia treatment has been commenced
- If no improvement in VA beyond ≥0.500 following six months of treatment, a review by consultant for repeat fundus check is arranged

In addition to examining patient outcomes, we analysed how long an average patient journey was; from the first appointment to the date of discharge, with amblyopia treatment being completed during this period.

We also examined the percentage of patients who achieved correction of their amblyopia at their first review visit (six to eight week appointment). This allowed us to determine whether the six to eight week review appointment could be extended, which in turn could prove to have good financial and capacity benefits.

## Methods

A retrospective database review was conducted to identify patients who fit the inclusion criteria.

### Inclusion criteria

Patients who had undergone treatment for anisometropic, strabismic or mixed amblyopia, and had been discharged including those with a logMAR VA assessment during 31.8.16 and 1.9.19. Patients may have had any type of occlusion treatment; patching, atropine, optical penalisation, fablon or a combination.

### Exclusion criteria

Patients with stimulus deprivation or ametropic amblyopia were excluded, as were community follow ups due to access to data.

Data collected included:

- Age
- VA at presentation and discharge (logMAR)
- Amblyopia type
- Treatment type
- Length of whole episode
- Adverse events
- Proportion of vision change
- Proportion of those who were able to discontinue occlusion at the first six to eight week return visit

T-tests were carried out to determine whether mean final VA outcomes for each type of amblyopia were statistically significant to mean final VA outcomes achieved in 2011–12.

We separated data into 3 groups for analysis based on the final VA of the amblyopic eye, in accordance with the previous audit:

- ≤0.250
- 275 – 0.500
- ≥0.525

In line with the previous audit, to analyse proportion of VA change from pre-occlusion to post-occlusion, we assigned numbers according to this change (Stewart et al, 2003):

- Negative number = VA in the amblyopic eye had reduced or amblyopic eye had remained stable while the fellow eye had improved. For example a pre-occlusion VA of 0.200 and 0.600 and a post-occlusion VA of 0.200 and 0.800 or a pre-occlusion VA of 0.200 and 0.600 to a post-occlusion VA of 0.000 and 0.600.
- 0 = no change to VA with occlusion. For example, if a patient had a pre-occlusion VA of 0.200 and 0.600 and the post-occlusion VA remained at 0.200 and 0.600.
- 1 = the amblyopic deficit was fully corrected and the final VAs were equal i.e. the optimum outcome. For example, if a patient had a pre-occlusion VA of 0.200 and 0.600 and post-occlusion VA of 0.200 and 0.200.
- >1 = VA of the once amblyopic eye had increased to a level higher than that of the fellow eye. For example, a pre-occlusion VA of 0.200 and 0.600 and a post-occlusion VA of 0.200 and 0.100.

During analysis we used slightly different category groups for the scores assigned, as there appeared to be some cross-over in the categories used in the previous audit.

## Results

The orthoptic database identified 1,100 patients who were discharged between 31.8.16 and 1.9.19. Of these, 926 were excluded due to not fitting the inclusion criteria, which left 174 patients to be included in the analysis.

There were 58 anisometropic, 64 strabismic and 52 mixed amblyopes.

The mean age at presentation was 55 months (SD 19 months, range 2 – 97 months). In the previous audit, 373 patients were included in the analysis. There were 153 anisometropic, 110 strabismic and 110 mixed. The mean age at presentation was 46 months, range 2–124 months).

### VA outcomes

Unpaired T-tests show that for each type of amblyopia, there was no significant difference in VA outcomes between the current audit and the previous audit (anisometropic amblyopia p = 0.317, strabismic amblyopia p = 0.438, mixed amblyopia p = 0.324) (Table 1).

**Table 1:** Final mean VAs (with SD and range) and T-test results for each type of amblyopia in the 2011–12 audit compared to the current audit.


AMBLYOPIA TYPE	PREVIOUS AUDIT (2011–12)			CURRENT AUDIT (31.8.16–1.9.19)			T-TEST
	
	MEAN FINAL VA (LOG UNITS)	SD	RANGE	MEAN FINAL VA (LOG UNITS)	SD	RANGE	

Anisometropic	0.243	0.355	0.125 to POL (perception of light)	0.220	0.109	–0.50 to 0.550	p = 0.317

Strabismic	0.332	0.190	0.050 to 1.250	0.363	0.275	0.025 to 1.250	p = 0.438

Mixed	0.422	0.272	0.125 to POL	0.367	0.280	0.025 to 1.550	p = 0.324


**Figure 1:**
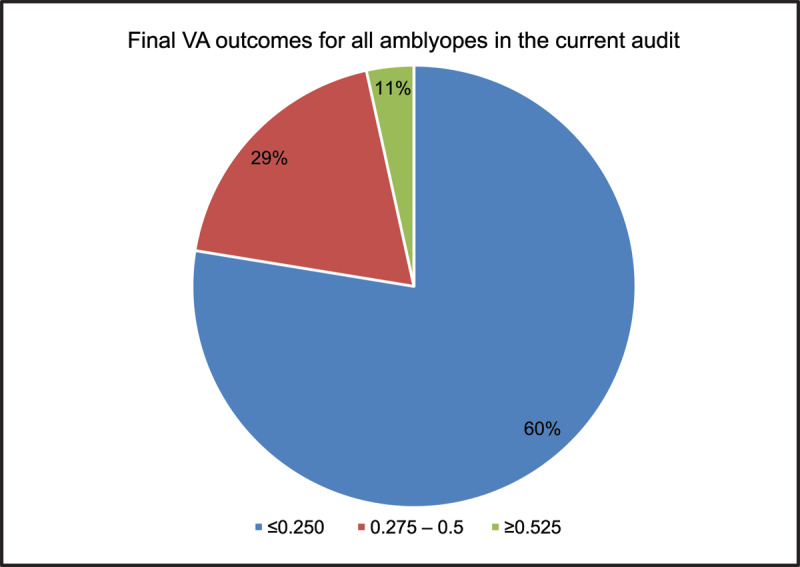
Final VA outcomes for all amblyopes classified into three VA groups.

For all amblyopes (anisometropic + strabismic + mixed), the majority of patients (60%) achieved a VA outcome of ≤0.250 (Table 2 and Figure 1). This is comparable to the previous audit where 59% of all amblyopes (anisometropic + strabismic + mixed) achieved a VA outcome of ≤0.250.

**Table 2:** Number of patients in each amblyopia group and their final VA outcomes classified into three VA groups in the 2011–12 audit compared to the current audit.


	PREVIOUS AUDIT (2011–12)			CURRENT AUDIT (PATIENTS DISCHARGED BETWEEN 31.8.16–1.9.19)		

Type of amblyopia	≤0.250 (or equal to)	0.275–0.500	≥0.525 (or equal to)	≤0.250 (or equal to)	0.275–0.500	≥0.525 (or equal to)

Anisometropic	117 (31%)	26 (7%)	10 (3%)	45 (26%)	11 (6%)	2 (1%)

Strabismic	53 (14%)	43 (12%)	14 (4%)	34 (20%)	20 (11%)	10 (6%)

Mixed	50 (13%)	31 (8%)	29 (8%)	25 (14%)	19 (11%)	8 (6%)

Total	220 (59%)	100 (27%)	53 (14%)	104 (60%)	50 (29%)	20 (11%)


### Proportion VA change

One hundred and thirty four patients used the same VA test throughout treatment and were included in this analysis.

**Figure 2:**
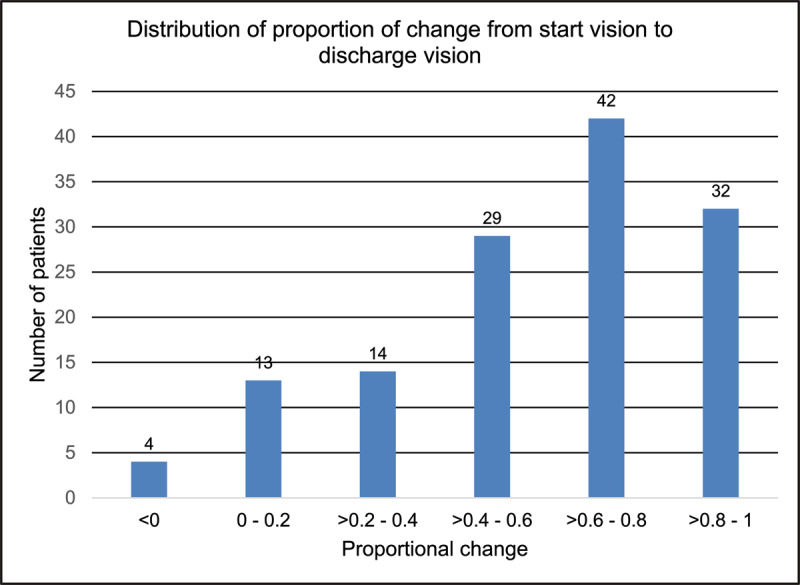
Number of patients in each proportion of VA change group in the current audit.

For all amblyopes, most patients (42 patients/31%) demonstrated proportional VA improvements >0.6–0.8, followed by 32 patients (24%) who demonstrated proportional VA improvements >0.8–1. Only four patients (3%) fall into the <0 group (Table 3 and Figure 2).

**Table 3:** Number of patients in each proportion of VA change group.


PROPORTION OF CHANGE GROUP	NUMBER OF PATIENTS

<0	4

0–0.2	13

>0.2–0.4	14

>0.4–0.6	29

>0.6–0.8	42

>0.8–1	32


**Figure 3:**
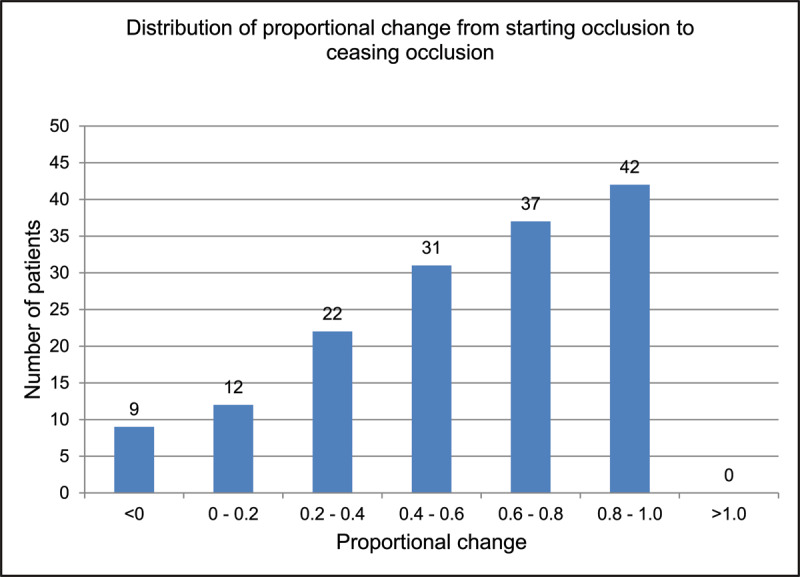
Number of patients in each proportion of VA change group in the previous audit (2011–12).

In the previous audit, 153 patients used the same VA test throughout treatment and were included in the proportion of change analysis. Most patients (42 patients/27%) demonstrated VA improvements 0.8–1, followed by 37 patients/24% who demonstrated VA improvements 0.6–0.8. Only nine patients (6%) fell into the <0 group (Figure 3).

### Episode Length

**Figure 4:**
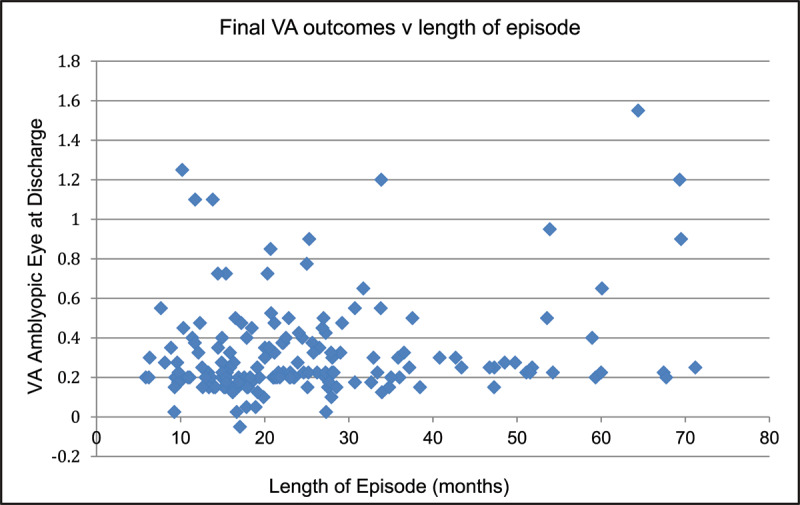
Final VA outcomes and length of whole episode for patients in the current audit.

The mean episode length, defined as date of first visit to date of discharge, was 25 months (SD 15 months, range 6–71 months) during which time treatment was commenced and completed (Figure 4).

Twenty-six patients (15%) completed treatment within a one-year period. The majority (108 patients/62%) had completed treatment within two years (Table 4).

**Table 4:** Cumulative number of patients completing their episode within one to six years in the current audit.


EPISODE LENGTH	CUMULATIVE NUMBER OF PATIENTS COMPLETING THEIR EPISODE

1 year	26 (15%)

2 years	108 (62%)

3 years	146 (84%)

4 years	156 (89%)

5 years	168 (97%)

6 years	174 (100%)


### First follow-up visit

Only 18/174 patients (10%) discontinued amblyopia treatment on their first (six to eight week) visit back due to the amblyopia deficit being corrected.

### Adverse events

There was one adverse event to atropine treatment in the audit cohort; it was documented that a patient’s asthma had worsened and so the child switched to patching. No adverse events were recorded with patching.

## Discussion

The results of this audit show that current mean VA outcomes are not statistically significantly different to previous mean VA outcomes for all amblyopes, showing that we are still achieving VA outcomes as satisfactory as those achieved in 2011–12. Most patients (60%) are still achieving a VA outcome of ≤0.250 logMAR (59% in the previous audit), which is considered a good level of VA as per standards set in the department and used for all internal audits (Taylor et al, 2010). This is followed by 29% who achieve a VA outcome between 0.275–0.5 logMAR (comparable to 27% in the previous audit) and only 11% who have a final VA outcome of ≥0.525 logMAR (comparable to 14% in the previous audit).

Assessing mean final Vas achieved gives some idea of the change in VA with treatment, however, to get a better understanding it is useful to analyse proportion of change. In keeping with the previous audit, proportion of change can only be analysed accurately when the VA test used is the same from the start of treatment to the end of treatment (Stewart et al., 2003). In this audit 134 patients were able to perform the crowded logMAR throughout their treatment length and were included in this calculation. The proportion of change analysis shows that we manage to improve most amblyopic eyes to a VA level almost equal to the vision of the fellow eye and a few patients (11 patients/8%) gain a completely equal level of vision. Only four patients (3%) did not achieve any VA improvement with treatment (comparable to 6% in the previous audit).

However, it is difficult to directly compare proportion of change between the two audits, as the proportion of change categories are labelled slightly differently due to some cross-over of categories in the previous audit. It is also worth considering that there isn’t a level of proportional change which is considered a “good” level, and more research might be useful.

Most of the patients (62%) completed their episode within a two-year period, during which time treatment was commenced, completed and the patient was discharged. In the previous audit, 92% of patients completed treatment within two years, however only the treatment length was analysed whereas the current audit analysed the length of the patient’s entire journey, so it is difficult to directly compare this aspect.

Examination into those patients in the current audit with longer episodes demonstrated a pattern of patients who were referred at a young age and amblyopia treatment not started straight away. This is often due to ability and co-operation. Alternatively, some complete their amblyopia treatment while still very young and are not discharged straight away. Other longer episodes can be explained by multiple missed appointments, more than one episode of treatment or parents who did not want to discontinue amblyopia treatment.

Only 10% of patients discontinued amblyopia treatment on their first visit back (six to eight weeks) due to correction of the amblyopia deficit. In this group of patients, 4% had been treated for anisometropic amblyopia, 3% for mixed amblyopia and 3% for strabismic amblyopia. This shows that it is acceptable to increase the appointment length time frame from six to eight weeks for each type of amblyopia, and this will not result in over treating a significant number of patients. No patients in this 10% came to any harm by having slightly more treatment than necessary and there were no cases of occlusion amblyopia. In any given timeframe of occlusion, it is not possible to determine exactly at which point the deficit has been corrected.

Increasing the follow-up visit would have a direct and significant impact on reducing unnecessary appointments, which is beneficial to the department financially and helps with capacity issues. It is also beneficial for parents/guardians who may require time away from work to attend appointments and often have to pay travel and parking costs, as well as having environmental benefits in reducing the carbon footprint.

There was one adverse event in our cohort, and this was with the use of atropine, which was reported to have worsened a patient’s asthma. In this case the treatment was switched to a patch. Adverse events can occur for both treatment options (Osborne et al 2018) and it is important that the discussion regarding this remains in the amblyopia treatment protocol so that parents can make an informed decision on treatment choice.

There were significantly fewer patients included in the current audit in comparison to the previous audit. This was because the current audit analysed patients discharged between two dates, whereas the previous audit analysed patients who had commenced amblyopia treatment between two dates.

During the dates of the current audit, there were also significantly more patients who were followed-up in community and therefore excluded, due to the opening of a new outreach clinic.

## Conclusion

This audit shows that our current VA outcomes following amblyopia treatment are similar to those achieved in 2011–12, and that the current protocol remains efficient guidance for the successful treatment of amblyopia. The audit shows that after commencing amblyopia treatment, the first follow-up visit can be safely extended from six to eight weeks without over treating a significant number of patients. We have implemented this change in the NEC and the first follow-up visit is now 14–16 weeks (three to four months). This was particularly helpful during and following the Covid-19 pandemic where orthoptic appointment capacity was significantly affected. Future departmental amblyopia audits should aim to determine whether this increased follow-up time is appropriate and continuous to be clinically justified. The next amblyopia outcome audit should aim determine the percentage of patients who discontinue treatment at this point and will allow us to decide whether this is an appropriate follow-up time frame.
